# LncRNA UCA1 is necessary for TGF‐β‐induced epithelial–mesenchymal transition and stemness via acting as a ceRNA for Slug in glioma cells

**DOI:** 10.1002/2211-5463.12533

**Published:** 2018-10-18

**Authors:** Zongping Li, Hongyuan Liu, Qi Zhong, Jian Wu, Zhi Tang

**Affiliations:** ^1^ Department of Neurosurgery Mianyang Central Hospital China; ^2^ Department of Neurosurgery Yanting County People's Hospital Mianyang China

**Keywords:** ceRNA, glioma, lncRNA, stemness, TGF‐β, UCA1

## Abstract

The 5‐year survival rate of patients with glioma is < 5%, and therefore there is an urgent need to find novel potential targets for facilitating its diagnosis and treatment. The long non‐coding RNA (lncRNA) UCA1 has been shown to promote the proliferation and invasion of cervical cancer cells through regulating miR‐206 expression, but the involvement of UCA1 in regulating the stemness and epithelial–mesenchymal transition (EMT) of glioma cells is unknown. Here, we report that the expression of UCA1 is significantly increased by transforming growth factor‐β (TGF‐β) treatment in glioma cells and is greater in glioma tissues than in normal adjacent tissues. Additionally, TGF‐β induced EMT and the stemness of glioma cells, whereas knockdown of lncRNA UCA1 attenuated these two processes and their enhancement by TGF‐β. Mechanistically, knockdown of UCA1 decreased Slug expression by acting as a competitive endogenous RNA (ceRNA) through competitive binding with miR‐1 and miR‐203a; this effect was further evidenced by the fact that transfection with miR‐1 or miR‐203a inhibitors abrogated the effects of UCA1 knockdown on Slug expression, and UCA1 colocalized with miR‐1 and miR‐203a in glioma tissues. Notably, ectopic expression of Slug rescued the attenuation of UCA1 knockdown on EMT and the stemness of glioma cells. These results indicate that UCA1 may act as a ceRNA to promote Slug expression, which underlies TGF‐β‐induced EMT and stemness of glioma cells.

**Abbreviations**
3′UTR: 3′‐untranslated region
ALDH1: aldehyde dehydrogenase 1
ceRNA: competitive endogenous RNA
CSC: cancer stem cell
EMT: epithelial–mesenchymal transition
lncRNA: long non‐coding RNA
NC: negative control
qRT‐PCR: quantitative real‐time PCR
RIP: RNA immunoprecipitation
RNA‐FISH: RNA fluorescence *in situ* hybridization
siRNA: small interfering RNA
TGF‐β: transforming growth factor‐β

Glioma is one of the most common primary tumors in human brain with invasive growth, and the 5‐year survival rate of patients is still < 5% even with comprehensive treatment such as surgery, radiation and chemotherapy 1. Therefore, it is remains urgent to find novel potential targets for facilitating the diagnosis and treatment of glioma.

Cancer stem cells (CSCs) or cells with stemness are a small proportion of the cells in tumor tissues that have the potential of self‐renewal and undirectional differentiation 2. Although of low content in tumors, they hold strong tumorigenicity and have been regarded as the root of tumor metastasis and chemoresistance 3. However, the mechanisms underlying CSC progression are still unclear. Long non‐coding RNAs (lncRNAs) are a class of transcripts longer than 200 nucleotides with limited protein‐coding ability that have been confirmed to play critical roles in various aspects of tumor progression, such tumor angiogenesis 4 and metastasis 5. LncRNA UCA1 has been shown to promote the proliferation and invasion of cervical cancer cells through regulating miR‐206 expression 6. A recent study has indicated that lncRNA UCA1 modulates glioblastoma‐associated stromal cell‐mediated glycolysis and invasion of glioma cells 7. However, the roles and related mechanisms of UCA1 in regulating the stemness and epithelial–mesenchymal transition (EMT) of glioma cells have not been revealed.

Transforming growth factor‐β (TGF‐β), as a major inducer of EMT, could promote the invasion and metastasis in non‐small cell lung cancer progression 8. A recent study has demonstrated that TGF‐β can increase the stemness of MiaPaCa‐2 cells 9. Here, we investigated the roles and related mechanisms of TGF‐β in regulating EMT and the stemness of glioma cells. We found that lncRNA UCA1 expression was induced by TGF‐β in glioma cells. Notably, TGF‐β facilitated EMT and the stemness, and this effect was attenuated by UCA1 knockdown. Furthermore, we identified that lncRNA UCA1 promoted the expression of Slug, a downstream effector of TGF‐β, by acting as a competitive endogenous RNA (ceRNA) through competitively binding to miR‐1 and miR‐203a. Importantly, overexpression of Slug abrogated the attenuation of UCA1 knockdown on the EMT and stemness of glioma cells, and their expression exhibited a positive correlation in glioma tissues. These results suggest that lncRNA UCA1 functions as a critical regulator of EMT and the stemness of glioma cells, which could be a potential target for glioma.

## Materials and methods

### Clinical samples

Thirty‐three glioma and normal adjacent tissue samples were obtained from the Mianyang Central Hospital between September 2015 and February 2018. The experiments were undertaken with the understanding and written consent of each subject. The study methodologies conformed to the standards set by the Declaration of Helsinki. Written informed consent from all patients and approval of the hospital ethics review committees were obtained.

### Cell culture and reagents

Human glioma cell lines U87 and U251 were purchased from the cell bank of Chinese Academy of Sciences (Shanghai, China). Both of them were cultured in RPMI 1640 medium (Thermo Fisher Scientific, Waltham, MA, USA), supplemented with 10% FBS (Thermo Fisher Scientific) under a humidified atmosphere with 5% CO_2_ at 37 °C. Recombinant Human TGF‐β1 was purchased from Peprotech (cat. no. 100‐21, Rocky Hill, NJ, USA). The plasmid SlugMyc_pcDNA3 was used to transfect into cells to upregulate Slug expression, and purchased from Addgene (cat. no. 31698, Cambridge, MA, USA).

### Quantitative real‐time PCR

Quantitative real‐time PCR (qRT‐PCR) was performed to examine the mRNA level of gene transcripts. Total RNA from cells or tissues was extracted using TransZol Up (Transgen Biotech, Beijing, China) according to the manufacturer's recommendation. First‐strand cDNA was synthesized using SuperScript™ IV First‐Strand Synthesis System (cat. no. 18091050; Thermo Fisher Scientific) following the manufacturer's protocols. Then cDNA was used to perform qRT‐PCR on the StepOne Plus PCR system with TransStart Green qPCR SuperMix (Transgen Biotech). Glyceraldehyde 3‐phosphate dehydrogenase served as an internal reference. The $2^{-\Delta \Delta C_{t}}$ method was used to quantify the relative expression level of transcripts.

### Western blot

For detailed procedure, refer to the previous study 10. Briefly, cells were lysed using RIPA protein extraction reagent (Beyotime, Shanghai, China) supplemented with a protease inhibitor cocktail (Roche, Basel, Switzerland). Protein concentration was measured using the Bio‐Rad protein assay kit (Bio‐Rad, Beijing, China). Approximately 30 μg of protein extract was separated by 10% SDS/PAGE, then transferred to nitrocellulose membrane (Sigma‐Aldrich, St Louis, MO, USA) and incubated with the primary antibodies against E‐cadherin (cat. no. 20874‐1‐AP), Slug (cat. no. 12129‐1‐AP), Nanog (cat. no. 14295‐1‐AP), aldehyde dehydrogenase 1 (ALDH1) A1 (cat. no. 15910‐1‐AP), Dicer (cat. no. 20567‐1‐AP) and β‐actin (cat. no. 60008‐1‐Ig), which were purchased from Proteintech. The secondary antibodies (cat. no. A0216 and cat. no. A0239), horseradish peroxidase‐conjugated, were purchased from Beyotime. An Enhanced Chemiluminescence Plus kit (cat. no. YP0071; Yi Fei Xue Biotechnology, Nanjing, China) was used to develop an image in a Tanon 5200 machine (Tanon, Shanghai, China).

### Transfection of siRNA, miRNA mimics and inhibitor

When cell confluency reached 60–80%, cells were transfected with a final concentrations of 50 nm miR‐1 or miR‐203a (mimics or inhibitor), 50 nm small interfering RNA (siRNA) and 50 nm negative control (NC), which were synthesized by GenePharma (Shanghai, China), and plasmids using Lipofectamine 2000 Reagent (Thermo Fisher Scientific) following the manufacturer's recommendation.

### Luciferase reporter analysis

The sequences of lncRNA UCA1 and the 3′ untranslated region of Slug were inserted into pMIR‐Report, denoted as Luc‐UCA1‐wt and Luc‐Slug‐3′UTR, respectively. Sequences of UCA1 with a mutated binding site of miR‐1 or miR‐203a, or mutated binding sites of both miR‐1 and miR‐203a were obtained using Fast Mutagenesis Kit V2 (Vazamy, Nanjing, China) according to the manufacturer's instruction, and named Luc‐UCA1‐mut‐1, Luc‐UCA1‐mut‐203a and Luc‐UCA1‐mut, respectively. The binding sites of either miR‐1 or miR‐203a, and both of them on Slug 3′‐untranslated region (3′UTR) were mutated in the same way, and denoted Luc‐Slug‐3′UTR‐mut‐1, Luc‐Slug‐3′UTR‐mut‐203a and Luc‐Slug‐mut, respectively. For miRNAs targeting confirmation, Luc‐UCA1‐wt, Luc‐UCA1‐mut‐1 or Luc‐UCA1‐mut‐203a was cotransfected with miRNA mimics, inhibitor or NC, and β‐gal plasmid into glioma cells using Lipofectamine 2000 Reagent. For confirming the interaction between UCA1 and Slug 3′UTR, Luc‐Slug‐3′UTR, Luc‐Slug‐3′UTR‐mut‐1, Luc‐Slug‐3′UTR‐203a or Luc‐Slug‐3′UTR‐mut was cotransfected with siRNA against UCA1, or cotransfected with Luc‐UCA1‐wt, Luc‐UCA1‐mut‐1, Luc‐UCA1‐mut‐203a or Luc‐UCA1‐mut; 72 h later, cells were lysed with Reporter lysis buffer (cat. no, E397A; Promega Corp., Madison, WI, USA) and luciferase activity was measured with the VivoGlo Luciferin kit (cat. no., P1041; Promega Corp.) using a luminometer (Thermo Fisher Scientific) and normalized to β‐gal activity.

### RNA immunoprecipitation assay

RNA immunoprecipitation (RIP) assay was performed using a Magna RIP™ RNA‐Binding Protein Immunoprecipitation Kit (cat. no. 17‐700; Merck, Billerica, MA, USA) following the manufacturer's protocol. Briefly, glioma cells were transfected with Luc‐UCA1‐wt, Luc‐UCA1‐mut‐1 or Luc‐UCA1‐mut‐203a and lysed with lysis buffer. Cells extract was then incubated with binding buffer containing Protein A/G Agarose Resin conjugated with human anti‐Ago2 antibody (cat. no. ab32381; Abcam, Cambridge, MA, USA) at 4 °C overnight, followed by centrifugation and incubating with protease K to dissociate the Ago2‐RNA complex from the beads. qRT‐PCR was performed to detect miR‐1 and miR‐203a level.

### RNA fluorescence *in situ* hybridization

RNA fluorescence *in situ* hybridization (RNA‐FISH) for miR‐1 or miR‐203a and lncRNA UCA1 was performed on frozen slices of glioma tissue. This experiment was performed by Shanghai Gefan Biotechnology Co., Ltd. (Shanghai, China) Probes directed against miR‐1, miR‐203a and lncRNA UCA1 were synthesized by GenePharma (Shanghai, China). They were observed with confocal microscopy.

### Cell spheroid formation assay

Glioma cell spheroid formation was performed under anchorage‐independent conditions in methylcellulose (Sigma‐Aldrich). Briefly, glioma cells with different treatment were digested with Trypsin/EDTA (Sigma‐Aldrich), and then cultured in DMEM‐F12 medium supplemented with B27 (20 ng·mL^−1^) and epidermal growth factor (10 ng·mL^−1^) in non‐adherent 24‐well plates at 500 cells/well. After 8 days, spheres > 50 μm were counted. This experiment was performed in triplicate and repeated at least three times independently.

### ALDH1 activity assay

ALDH1 activity was assayed by ALDEFLUOR™ Kit (cat. no. KA3742; Stemcell Technologies, Vancouver, BC, Canada) following the standard procedure.

### Statistical analysis

All data were obtained from at least three independent experiments (*n* ≥ 3), and presented as the mean ± standard deviation (SD). The difference was assessed using one‐way ANOVA with the Tukey–Kramer *post hoc* test, and *P* < 0.05 was considered significant.

## Results

### TGF‐β treatment induced EMT and the stemness of glioma cells in a concentration‐dependent manner

**Figure 1:**
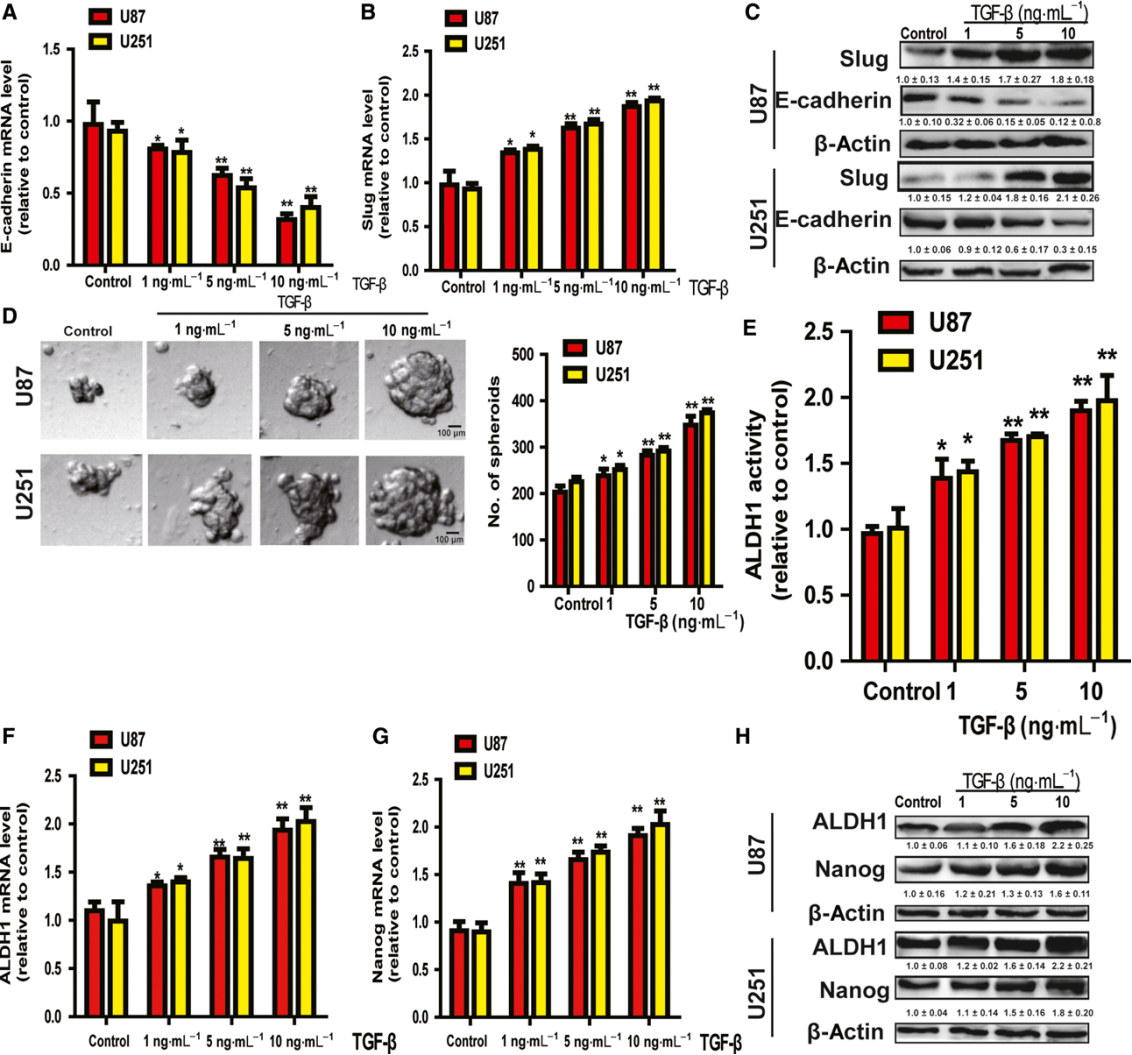
TGF‐β treatment induced EMT and the stemness of glioma cells in a concentration‐dependent manner. (A–C) The mRNA and protein level of E‐cadherin and Slug were examined in glioma cells treated with different concentrations of TGF‐β as indicated. (D) The capacity for cells spheroid formation was determined on the same glioma cells analyzed in (A). (E) ALDH1 activity was measured on the same glioma cells analyzed in (A). (F–H) The mRNA and protein levels of ALDH1 and Nanog were determined in cells depicted in (A). Scale bar, 100 μm. The difference was assessed using one‐way ANOVA with the Tukey–Kramer *post hoc* test. Data are presented as the mean and SD; *n* ≥ 3, *P < 0.05, **P < 0.01 *vs* Control. The densitometric analysis values are the means of three independent blots and representative blots are shown.

Firstly, we confirmed that TGF‐β could induce the EMT process. As shown in Fig. 1A–C, the expression of the epithelial marker E‐cadherin was significantly decreased in glioma cells with TGF‐β treatment in a concentration‐dependent manner, while the expression of the mesenchymal marker Slug, which was also the downstream effector of TGF‐β signaling 11, displayed the opposite effect. Furthermore, the stemness of glioma cells was enhanced by TGF‐β treatment in a concentration‐dependent manner, characterized as the promotion of spheroid formation (Fig. 1D) and upregulation of ALDH1 activity (Fig. 1E). Additionally, the expression of stemness markers (Nanog and ALDH1) was increased by TGF‐β treatment too (Fig. 1F–H). Taken together, these results indicate that TGF‐β could indeed induce EMT and the stemness of glioma cells.

### LncRNA UCA1 was upregulated by TGF‐β treatment and responsible for TGF‐β‐induced effects

**Figure 2:**
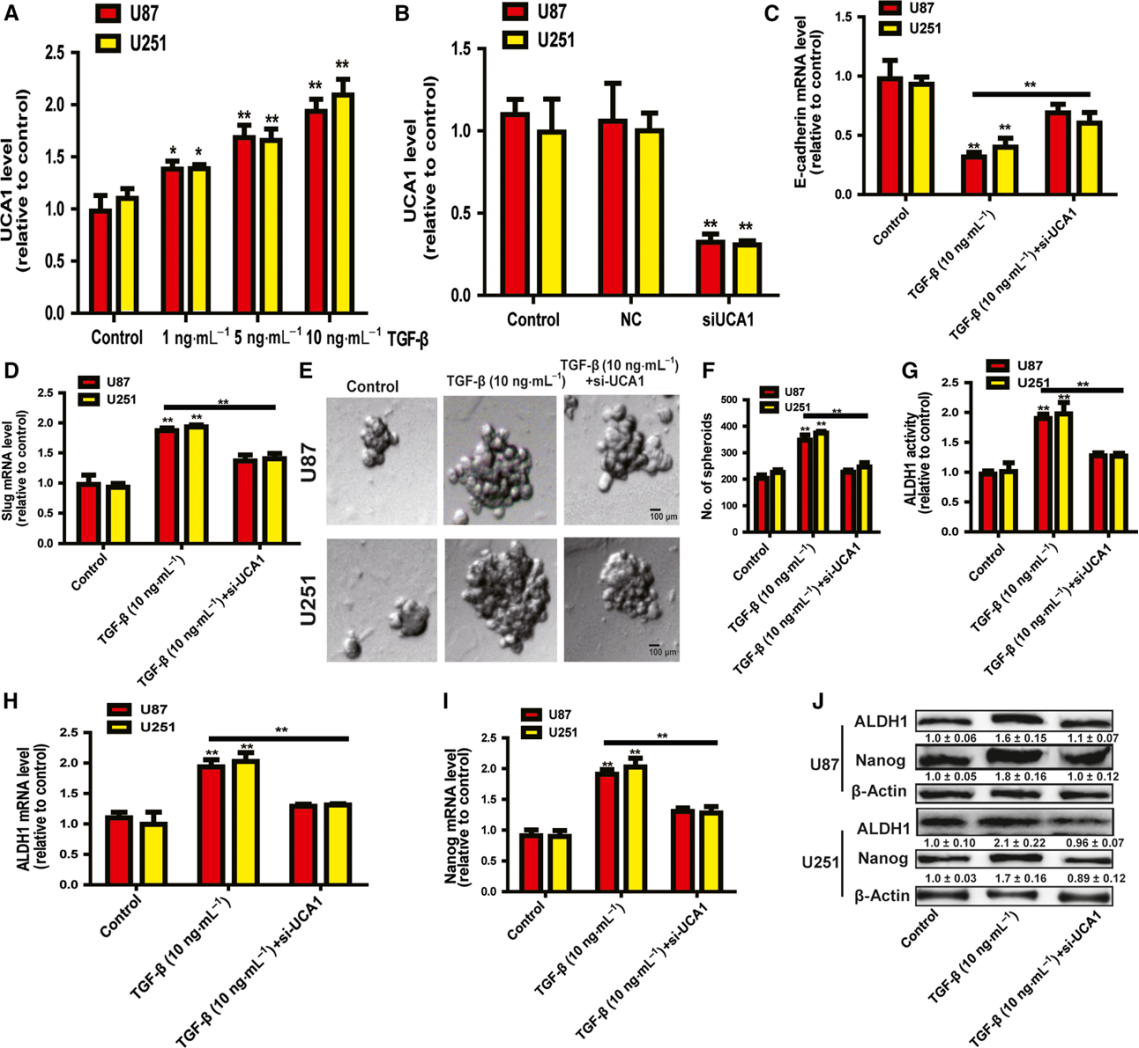
The level of lncRNA UCA1 was upregulated by TGF‐β treatment and responsible for TGF‐β‐induced effects. (A) LncRNA UCA1 level was measured in glioma cells with different concentrations of TGF‐β as indicated. (B) The knockdown efficiency of siUCA1 was confirmed by qRT‐PCR. (C,D) The mRNA level of E‐cadherin and Slug was examined in glioma cells with or without TGF‐β treatment plus si‐UCA1. (E,F) The capacity for cell spheroid formation was performed on the same glioma cells analyzed in (C). (G) ALDH1 activity was measured on the same glioma cells analyzed in (C). (H–J) The mRNA and protein level of ALDH1 and Nanog were examined on the same glioma cells analyzed in (B). Scale bar, 100 μm. The difference was assessed using one‐way ANOVA with the Tukey–Kramer *post hoc* test. Data are presented as the mean and SD; *n* ≥ 3, *P < 0.05, **P < 0.01 *vs* Control. The densitometric analysis values are the means of three independent blots and representative blots are shown.

We further investigated whether lncRNA UCA1 was involved in TGF‐β‐induced effects in glioma cells. Firstly, we found that UCA1 expression was induced by TGF‐β in a concentration‐dependent manner (Fig. 2A). The knockdown efficiency and specificity of siUCA1 were confirmed by qRT‐PCR in glioma cells (Fig. 2B). As expected, knockdown of UCA1 attenuated the promotive effects of TGF‐β on the EMT process in glioma cells (Fig. 2C,D). In addition, the enhancement of the stemness of glioma cells mediated by TGF‐β was abrogated by UCA1 knockdown, evident by the rescue of sphere formation (Fig. 2E,F), ALDH1 activity (Fig. 2G) and the expression of stemness markers (Fig. 2H–J). Our results suggest that lncRNA UCA1 is responsible for TGF‐β‐induced promotion of the EMT and stemness of glioma cells.

### Knockdown of lncRNA UCA1 attenuated EMT and the stemness of glioma cells

**Figure 3:**
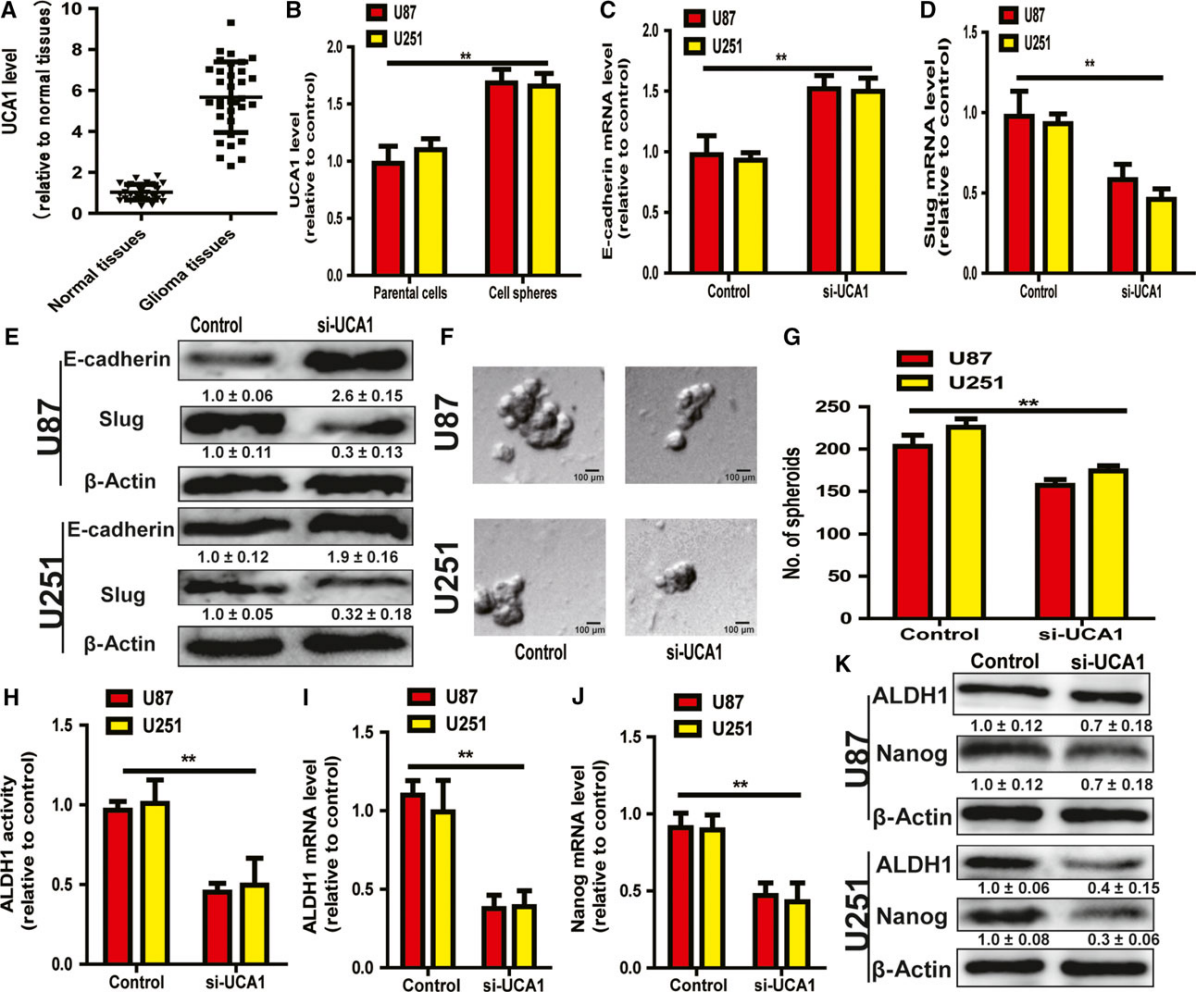
Knockdown of lncRNA UCA1 attenuated EMT and the stemness of glioma cells. (A) The level of lncRNA UCA1 was determined in glioma and normal adjacent tissues. (B) The level of lncRNA UCA1 was determined in glioma cells and cells spheres. (C–E) The mRNA and protein levels of E‐cadherin and Slug were examined in glioma cells with or without UCA1 knockdown. (F,G) The capacity of cell spheroid formation was evaluated in cells depicted in (C). (H) ALDH1 activity was analyzed in the same glioma cells as in (C). (I–K) The mRNA and protein levels of ALDH1 and Nanog were determined in the same glioma cells analyzed in (C). Scale bar, 100 μm. The difference was assessed using one‐way ANOVA with the Tukey–Kramer *post hoc* test. Data are presented as the mean and SD; *n* ≥ 3, **P < 0.01 *vs* Control. The densitometric analysis values are the means of three independent blots and representative blots are shown.

Based on the above results, we deduced that lncRNA UCA1 alone had the promotive effects on the EMT and stemness in glioma cells. Firstly, the UCA1 level was determined in glioma and normal tissues, and we found that it was remarkably upregulated in glioma tissues (Fig. 3A). The UCA1 level was further measured in glioma cells and cell spheres, and a qRT‐PCR result showed that it was remarkably upregulated in glioma cell spheres compared with the parental cells (Fig. 3B). As expected, knockdown of UCA1 attenuated the EMT process in glioma cells characterized as an increase of the epithelial maker E‐cadherin expression, and decrease of the mesenchymal marker Slug expression (Fig. 3C–E). What is more, knockdown of UCA1 decreased the capacity for cell sphere formation, shown as a decrease of spheres size and number (Fig. 3F,G). Additionally, both ALDH1 activity (Fig. 3H) and the expression of stemness markers (ALDH1 and Nanog) (Fig. 3I–K) were reduced by UCA1 knockdown. Therefore, our results demonstrate that lncRNA UCA1 could indeed promote the EMT and stemness of glioma cells.

### LncRNA UCA1 promoted Slug expression through regulating miRNA activity

**Figure 4:**
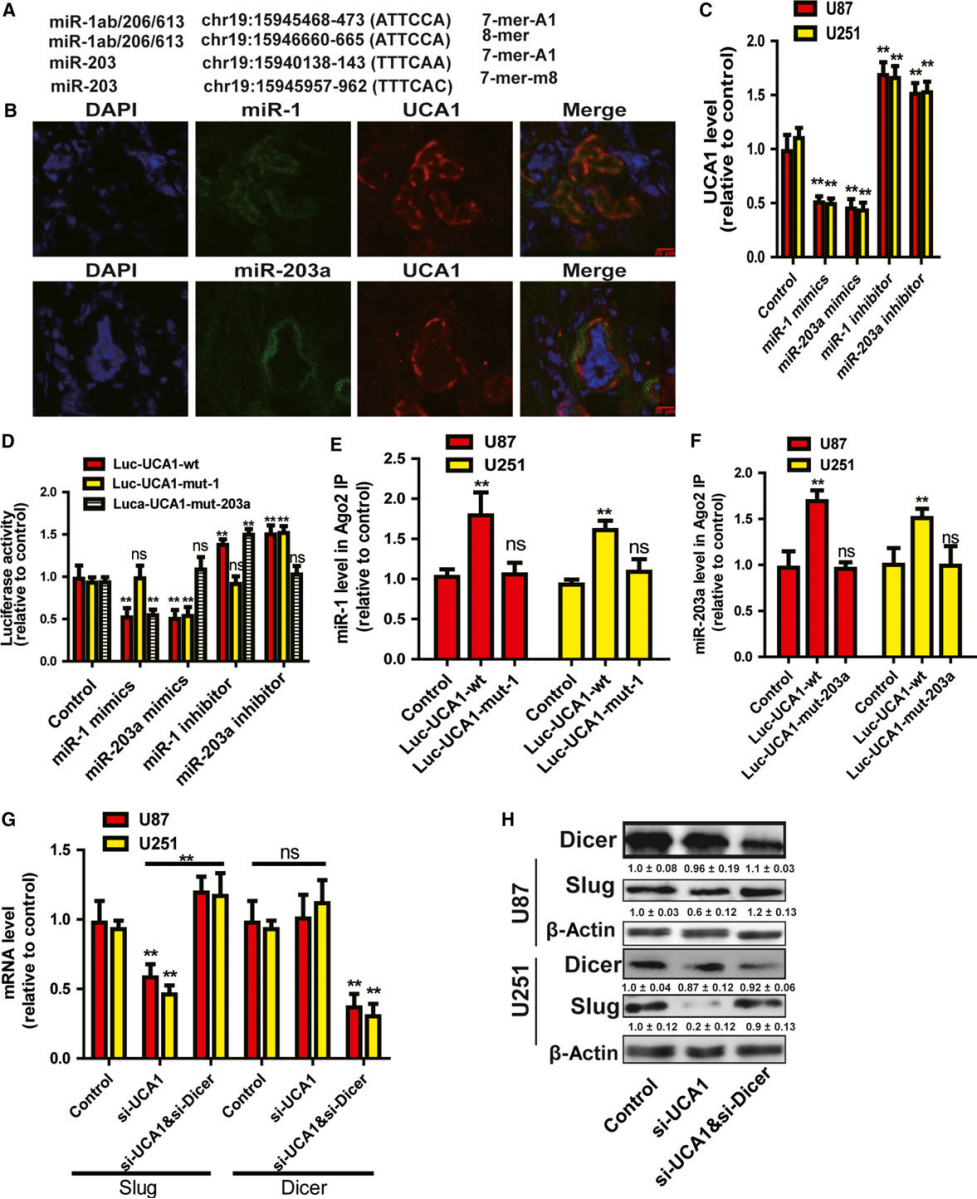
LncRNA UCA1 promoted Slug expression through regulating miRNA activity. (A) A schema showing the binding sites for miR‐1 and miR‐203a on UCA1. (B) RNA‐FISH was performed in glioma tissues, and results showed that both miR‐1 and miR‐203 were colocalized with UCA1 in the cytoplasm, scale bar 20 μm. (C) UCA1 level was determined in glioma cells transfected with miRNA (miR‐1 and miR‐203) mimics or inhibitor. (D) Luc‐UCA1‐wt, Luc‐UCA1‐mut‐1 or Luc‐UCA1‐mut‐203 was cotransfected with miRNA (miR‐1 and miR‐203) mimics or inhibitor; 72 h later, luciferase activity was measured. (E,F) qRT‐PCR was used to measure the abundance of miR‐1 or miR‐203a present in the Ago2‐IP material after a RIP assay in glioma cells transfected with Luc‐UCA1‐wt, Luc‐UCA1‐mut‐1 or Luc‐UCA1‐mut‐203a. (G,H) The mRNA level of Slug and protein of Slug and Dicer were determined in glioma cells with cotransfection of si‐UCA1 and si‐Dicer. The difference was assessed using one‐way ANOVA with the Tukey–Kramer *post hoc* test. Data are presented as the mean and SD; *n* ≥ 3, **P < 0.01 *vs* Control. The densitometric analysis values are the means of three independent blots and representative blots are shown.

As Slug is the critical downstream effector of TGF‐β signaling, we tried to explore the mechanisms underlying UCA1‐mediated regulation of Slug expression, and since plenty of evidence has confirmed the ceRNA activity of lncRNAs, we wondered whether lncRNA UCA1 could act as a ceRNA for Slug in glioma cells 5, 7, 12. Although many miRNAs were predicted by DIANA‐LncBase and miRcode analysis to have binding sites on UCA1, miR‐1 and miR‐203a attracted our attention because both of them were shown to suppress EMT by inhibiting Slug expression 13, 14 (Fig. 4A). To test our speculation, RNA‐FISH was performed to evaluate the localization of miR‐1, miR‐203a and UCA1 in glioma tissues; we found that both miR‐1 and miR‐203a were colocalized with UCA1 in cytoplasm of glioma cells (Fig. 4B). Then, glioma cells were transfected with miR‐1 or miR‐203a mimics or inhibitor, and qRT‐PCR results showed that overexpression of miR‐1 or miR‐203a significantly decreased UCA1 expression, while transfection of their inhibitors increased UCA1 expression (Fig. 4C). Furthermore, luciferase report analysis indicated that miR‐1 or miR‐203a mimics could decrease the luciferase activity of Luc‐UCA1‐wt, and miR‐1 or miR‐203a inhibitor increased the luciferase activity of Luc‐UCA1‐wt, whereas the luciferase activity of Luc‐UCA1‐mut‐1 was not affected by miR‐1 and Luc‐UCA1‐mut‐203a was unaffected by miR‐203a (Fig. 4D). Importantly, RIP analysis indicated that both miR‐1 and miR‐203a levels were enriched in RNA pulled down by ago2 in glioma cells transfected with Luc‐UCA1‐wt, but not in cells with Luc‐UCA1‐mut‐1 or Luc‐UCA1‐mut‐203a transfection (Fig. 4E,F). These results demonstrated that both miR‐1 and miR‐203a could definitely target lncRNA UCA1. Then we continue examining whether lncRNA UCA1 regulated Slug expression through miRNAs. SiRNA against Dicer, which is necessary for miRNA biogenesis, was cotransfected with UCA1 siRNA in glioma cells, and we found that knockdown of Dicer attenuated or even reversed the inhibition of UCA1 siRNA on Slug expression (Fig. 4G,H). Thus, these results indicate that lncRNA UCA1 could promote Slug expression through regulating miRNA activity.

### LncRNA UCA1 promoted Slug expression through the miR‐1 and miR‐203a binding sites on Slug 3′UTR and UCA1

**Figure 5:**
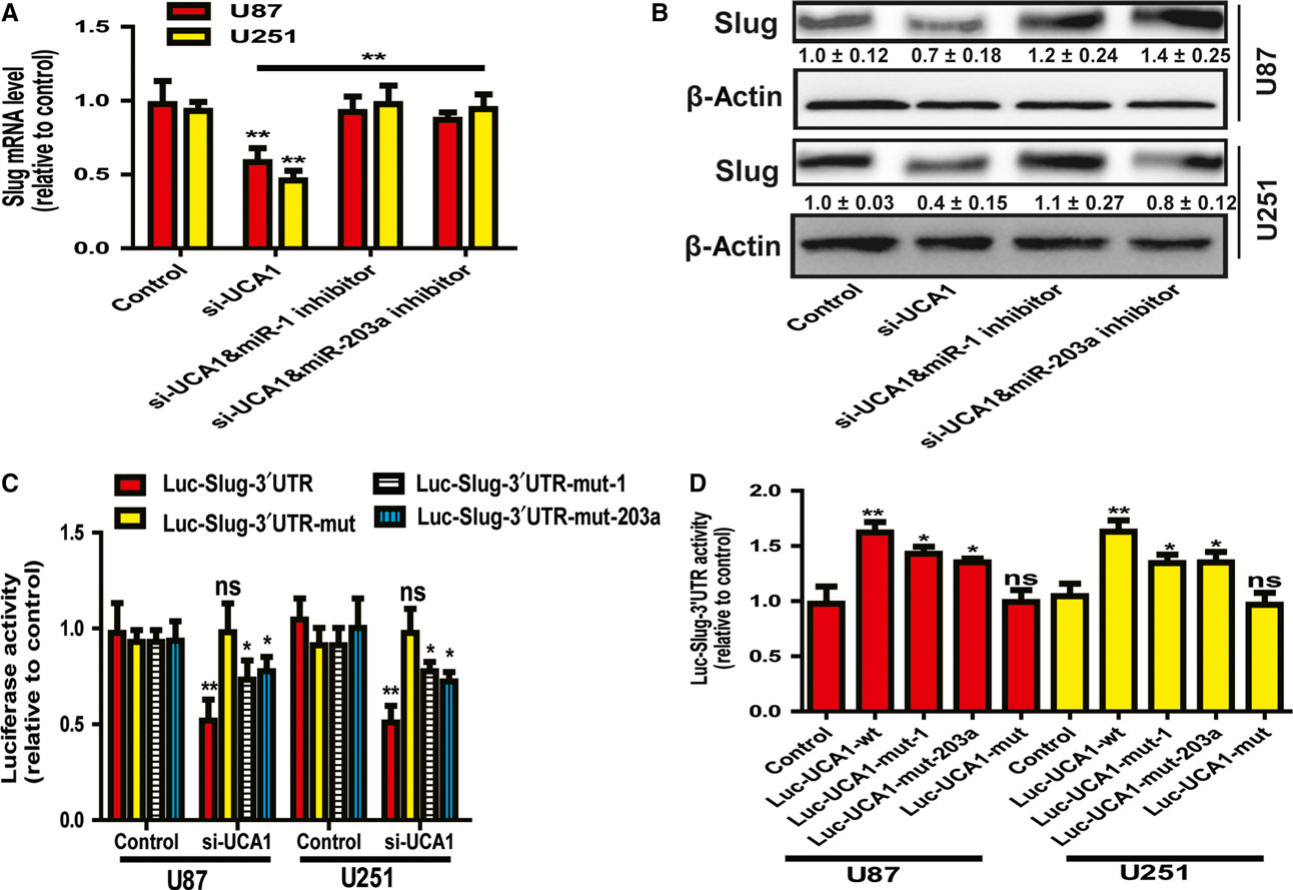
LncRNA UCA1 promoted Slug expression through the miR‐1 and miR‐203a binding sites on Slug 3′UTR and UCA1. (A,B) The mRNA and protein levels of Slug were examined in glioma cells transfected with si‐UCA1 as well as miRNA (miR‐1 or miR‐203a) inhibitor. (C) Luc‐Slug‐3′UTR, Luc‐Slug‐3′UTR‐mut, Luc‐Slug‐3′UTR‐mut‐1 or Luc‐Slug‐3′UTR‐mut‐203a was cotransfected with si‐UCA1 in glioma cells; 72 h later, the luciferase activity was measured. (D) Lucf‐Slug‐3′UTR was cotransfected with Luc‐UCA1‐wt, Luc‐UCA1‐mut, Luc‐UCA1‐mut‐1 or Luc‐UCA1‐mut‐203a in glioma cells, followed by detecting the luciferase activity after 72 h. The difference was assessed using one‐way ANOVA with the Tukey–Kramer *post hoc* test. Data are presented as the mean and SD; *n* ≥ 3, *P < 0.05, **P < 0.01 *vs* Control. The densitometric analysis values are the means of three independent blots and representative blots are shown.

To confirm that lncRNA UCA1 could act as a ceRNA for Slug, we needed to determine whether UCA1 regulates Slug expression through the binding sites of miR‐1 and miR‐203a on both Slug 3′UTR and UCA1 itself. As expected, transfection with miR‐1 or miR‐203a inhibitor rescued the decreased expression of Slug induced by UCA1 knockdown (Fig. 5A,B). Additionally, knockdown of UCA1 significantly decreased the luciferase activity of Luc‐Slug‐3′UTR, Luc‐Slug‐3′UTR‐mut‐1 and Luc‐Slug‐3′UTR‐mut‐203a, but not Luc‐Slug‐3′UTR‐mut, and the decreased extent of Luc‐Slug‐3′UTR was higher than Luc‐Slug‐3′UTR‐mut‐1 and Luc‐Slug‐3′UTR‐mut‐203a (Fig. 5C). Moreover, the luciferase activity of Luc‐Slug‐3′UTR was increased in glioma cells cotransfected with Luc‐UCA1‐wt, Luc‐UCA1‐mut‐1 or Luc‐UCA1‐mut‐203a, rather than with Luc‐UCA1‐mut (Fig. 5D). Notably, the extent of induction by Luc‐UCA1‐mut‐1 or Luc‐UCA1‐mut‐203a was weaker than that mediated by Luc‐UCA1‐wt. Altogether, our results show that lncRNA UCA1 could promote Slug expression by directly and competitively binding to miR‐1 and miR‐203a. Eventually, we had confirmed that lncRNA UCA1 could act as a ceRNA for Slug in glioma cells.

### LncRNA UCA1 attenuated EMT and the stemness of glioma cells dependent on Slug expression

**Figure 6:**
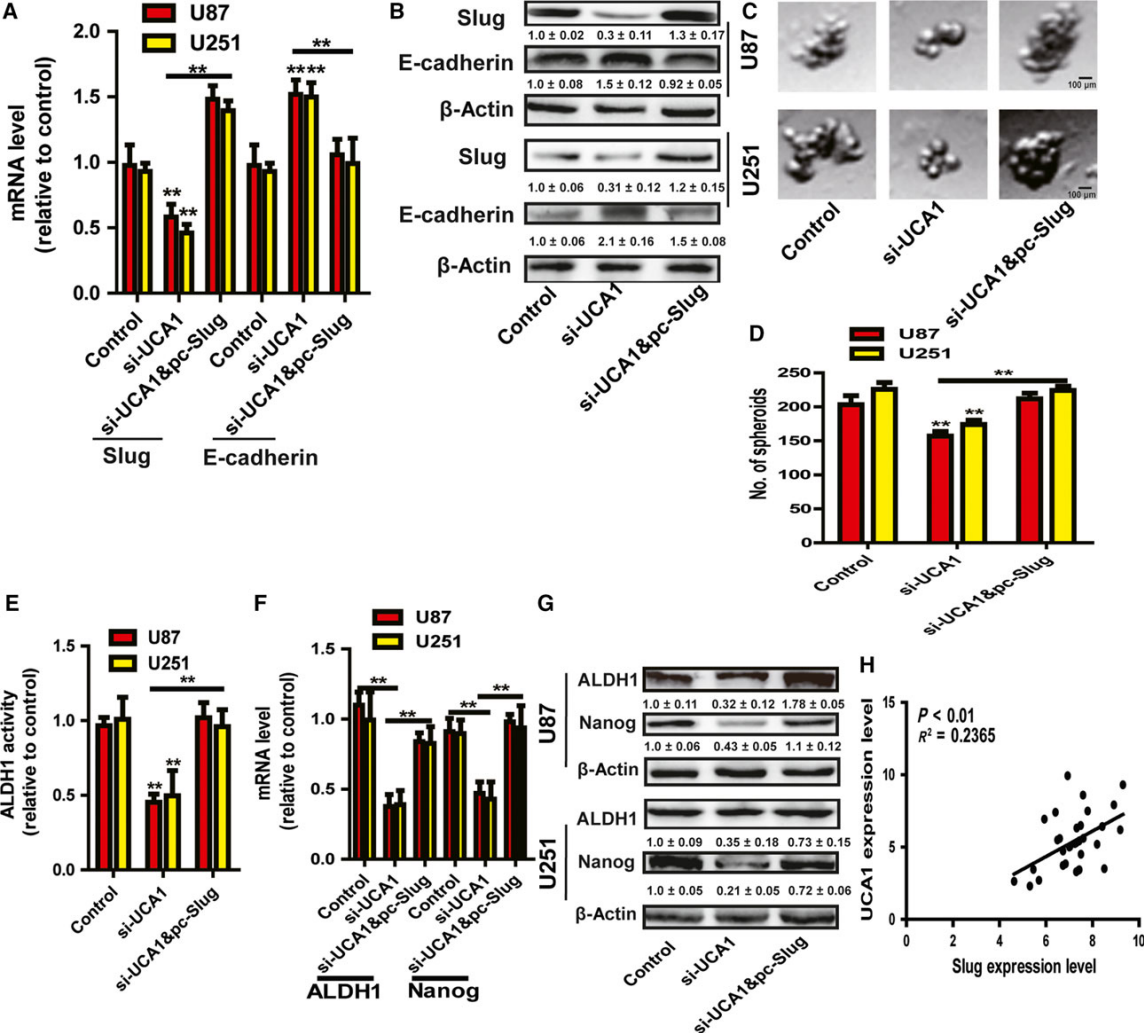
LncRNA UCA1 attenuated EMT and the stemness of glioma cells dependent on Slug expression. (A,B) The mRNA and protein levels of Slug and E‐cadherin were examined in glioma cells with UCA1 knockdown with or without Slug overexpression. (C,D) The capacity of cell spheroid formation was measured on the same glioma cells analyzed in (A). (E) ALDH1 activity was determined on the same glioma cells analyzed in (A). (F,G) ALDH1 and Nanog expression were evaluated on the same glioma cells analyzed in (A). (H) Slug and UCA1 expression displayed positive correlation in glioma tissues. Scale bar, 100 μm. The difference was assessed using one‐way ANOVA with the Tukey–Kramer *post hoc* test. Data were presented as the mean and SD; *n* ≥ 3, **P < 0.01 *vs* Control. The densitometric analysis values are the means of three independent blots and representative blots are shown.

Finally, we further determined whether lncRNA UCA1's effects were dependent on Slug expression. As shown in Fig. 6A,B, ectopic expression of Slug indeed rescued the decreased expression mediated by UCA1 knockdown, and also attenuated the promotion of UCA1 knockdown on E‐cadherin expression. Furthermore, the attenuation of UCA1 knockdown on the stemness of glioma cells was abrogated by Slug ectopic expression, evident by recovery of the capacity of cell spheroid formation (Fig. 6C,D), ALDH1 activity (Fig. 6E) and the expression of stemness markers (ALDH1 and Nanog) (Fig. 6F,G). Notably, the expression of lncRNA UCA1 and Slug was positively correlated in glioma tissues (Fig. 6H). Hence, these results suggest that lncRNA UCA1 attenuates EMT and the stemness of glioma cells dependent on expression of Slug, the downstream effector of TGF‐β signaling.

## Discussion

Cancer stem cells have been considered to be at the root of tumor progression and drug resistance, but the underlying mechanisms contributing to their development are still not well defined. In the present study, we showed that TGF‐β induced the expression of lncRNA UCA1, and promoted EMT and the stemness of glioma cells in a concentration‐dependent manner, and that this effect was rescued by UCA1 knockdown. Additionally, we found that the effects mediated by UCA1 were dependent on Slug, a downstream effector of TGF‐β signaling pathway. Although other signaling pathways could be activated by TGF‐β in glioma cells 15, and autocrine TGF‐β signaling maintains tumorigenicity of glioma‐initiating cells through Sry‐related HMG‐box factors 16 this is the first study showing the roles and related mechanisms of the TGF‐β–UCA1–Slug axis in the stemness and EMT of glioma cells.

The EMT process has been shown to be co‐adjusted by stemness in CSCs and tumor cells 2. Although the roles of TGF‐β in regulating the EMT process are well defined, the roles of TGF‐β in facilitating the stemness of glioma cells are still unclear. Here, we established the promotive roles of TGF‐β in the stemness of glioma cells, further confirming the interaction between the EMT process and the stemness. Previous studies have shown that lncRNA UCA1 contributes to drug resistance in tumors 17, 18; combining this with our results that UCA1 promotes the stemness of glioma cells, we speculate that lncRNA UCA1 could result in drug resistance in glioma cells via regulating the stemness of cells, which should be explored in future work.

Slug has been confirmed as the downstream effector of TGF‐β signaling pathway; here, we indicated that lncRNA UCA1 served as the downstream effector of TGF‐β and the upstream effector of Slug, respectively, which is consistent with recent studies in other cancers 14, 19, 20. Thus, we wonder whether the TGF‐β–UCA1–Slug regulatory axis is a common phenomenon in cancers. However, we must admit that the detailed mechanisms by which lncRNA UCA1 is regulated by TGF‐β are still unclear. Although recent work has shown that TGF‐β induced UCA1 expression in breast cancer through downstream Smad2/3 signaling 20, the detailed mechanisms underlying Smad2/3‐mediated regulating of UCA1 expression were not revealed. Notably, ceRNA activity of UCA1 in glioma cells has been indicated in the previous studies; for example, UCA1 targets miR‐122 to promote proliferation, migration and invasion of glioma cells 21. UCA1 could sponge miR‐204‐5p to promote migration, invasion and EMT of glioma cells via upregulation of ZEB1 22. And UCA1 interacts with miR‐182 to modulate glioma proliferation and migration by targeting iASPP 23. This work identified that UCA1 could exert its ceRNA activity through sponging miR‐1 and miR‐203a. These results suggest that UCA1 could act as a ceRNA for different transcripts through different miRNAs, and exert different functions in glioma cells.

Although more details should be clarified, we strongly believe the TGF‐β–UCA1–Slug regulatory axis plays important roles in regulating the stemness and EMT in glioma cells, and lncRNA UCA1 might be a potential target for glioma treatment or prognosis.

## Author contributions

ZL and ZT conceived and designed the project, ZL, HL and QZ acquired the data, ZL and JW analyzed and interpreted the data, and ZL and ZT wrote the paper.

## Conflict of interest

The authors declare no conflict of interest.
